# Eye Axial Length: Is There a Protective Link to Diabetic Retinopathy?

**DOI:** 10.7759/cureus.75712

**Published:** 2024-12-14

**Authors:** Aikaterini E Mouzaka, Aristeidis Chandrinos, Irini Chatziralli, Eleni Chatzichristou, Themistoklis K Gialelis

**Affiliations:** 1 Department of Ophthalmology, 251 Air Force General Hospital, Athens, GRC; 2 Optics and Optometry Division, Investigative Techniques in Optometry Research Group, Department of Biomedical Sciences, University of West Attica, Athens, GRC; 3 Optics and Optometry Sector, Department of Biomedical Sciences, Faculty of Health Sciences, University of West Attica, Athens, GRC; 4 2nd Department of Ophthalmology, National and Kapodistrian University of Athens School of Medicine, Athens, GRC

**Keywords:** axial length, diabetic retinopathy, myopia, ocular biomechanics, retinal vascular density

## Abstract

Diabetic retinopathy (DR) is a leading cause of vision impairment and blindness globally, particularly among working-age adults. As the prevalence of diabetes continues to rise, understanding factors that influence DR development and progression is increasingly important. Recent studies suggest a protective association between a longer axial length (AL) of the eye and the risk of DR, particularly in myopic individuals. This review explores the potential mechanisms underlying this relationship, including reduced retinal vascular density, altered retinal blood flow, and ocular biomechanics, which may collectively reduce the susceptibility of retinal tissues to hyperglycemic damage. However, limitations such as confounding factors, ethnic and genetic differences, and methodological challenges highlight the need for further research. This review aims to explore the relationship between AL and DR, examining the biological mechanisms that may underpin this association, summarizing the epidemiological evidence, discussing the clinical implications, and identifying directions for future research. Understanding the protective role of AL could have significant clinical suggestions, including more tailored screening intervals and personalized treatment approaches for DR. Future studies should focus on longitudinal analyses, mechanistic insights, and diverse populations to establish a clearer understanding of this relationship and its potential for novel therapeutic strategies.

## Introduction and background

Diabetic retinopathy (DR) is a major and growing public health concern and is recognized as one of the leading causes of visual impairment and blindness among working-age people worldwide [1]. According to the World Health Organization (WHO), the global prevalence of diabetes has almost quadrupled in the last 40 years, with a corresponding increase in the incidence of diabetic complications, including DR [2].

DR is characterized by progressive damage to the light-sensitive layer of the eye, the retina, due to chronic hyperglycemia. This damage can lead to severe visual impairment and eventually even blindness, in cases when left untreated [3]. Considering the increasing prevalence of diabetes and the significant impact of DR on quality of life, understanding the factors that influence the development and progression of this condition is of great importance [4].

The distance between the surface of the cornea and the retina is well known as the axial length (AL) of the eye. In ophthalmology, the AL of the eye is a critical anatomical parameter, affecting the refractive state of the eye as well as the overall ocular biomechanics. Recent studies have suggested that a longer AL length may have a protective effect on the development and the severity of DR. Thus, they raise interesting questions about the underlying mechanisms as well as the clinical implications of the AL length and DR relationship [5].

This review aims to explore the relationship between the AL and DR, examining the biological mechanisms that may underpin this association, summarizing the epidemiological evidence, discussing the clinical implications, and identifying directions for future research.

## Review

Overview of DR

DR is the leading cause of new cases of blindness in patients with diabetes mellitus [6]. This chronic, progressive, and sight-threatening retinal microvasculature disease is mainly associated with prolonged hyperglycemia in diabetes mellitus patients. It is one of the primary causes of vision impairment and blindness in working-age adults worldwide [6].

Non-proliferative DR (NPDR) and proliferative diabetic DR (PDR) are the two main stages of DR [7].

The early stage of DR, known as NPDR, only affects the retinal capillaries and does not involve the growth of new blood vessels. According to the extent observation of retinal damage, we could additionally classify NPDR into mild, moderate, and severe stages. Key features of NPDR include a) microaneurysms which are small bulges in retinal blood vessels that may often present as the first clinical sign of DR; b) intraretinal hemorrhages which are small, dot-like or blot-like hemorrhages within the retina; c) hard exudates which are lipid deposits resulting from leakage of serum lipoproteins from damaged blood vessels; d) retinal edema which is the swelling of the retina due to leakage of fluid from capillaries; e) cotton wool spots which are nerve fibers that appear as fluffy white patches on the retina; venous beading which is the irregular dilation and constriction of retinal veins and intraretinal microvascular abnormalities (IRMA) which represents the abnormal growth of small blood vessels within the retina [8].

PDR, as an advanced stage of DR, is characterized by the proliferation of new blood vessels on the retinal surface or the optic disc, a process known as neovascularization. These new vessels are fragile and prone to bleeding, leading to serious complications such as a) vitreous hemorrhage: bleeding into the vitreous gel, the transparent gel filling the eye, which can cause sudden vision loss; b) tractional retinal detachment: scar tissue formation from neovascularization that may pull on the retina, leading to detachment from the underlying tissue; c) neovascular glaucoma: new vessels growing on iris surface may block the normal flow of fluid runoff, leading to increased intraocular pressure and glaucoma; d) diabetic macular edema (DME), a complication of DR, that may occur at any stage being a significant cause of vision loss. DME is also characterized by fluid accumulation in the macula, the central part of the retina responsible for sharp and detailed vision [9].

Pathophysiology of DR

DR pathophysiology involves a complex interplay of both metabolic and biochemical pathways induced by chronic hyperglycemia. Several key processes contribute to the development and progression of DR.

High sugar levels within blood lead to overproduction of reactive oxygen species (ROS), causing oxidative stress [10]. The polyol pathway metabolizes excess glucose in the retina, converting it to sorbitol via aldose reductase. Accumulation of sorbitol within retinal cells leads to osmotic stress and cellular damage, contributing to vascular dysfunction [11]. 

Hyperglycemia activates protein kinase C (PKC), a group of enzymes that regulate specific cellular functions. PKC activation affects retinal blood flow, increases vascular permeability, and induces inflammation and neovascularization [12]. In addition, chronic hyperglycemia leads to the formation of advanced glycation end products (AGEs), which are proteins or lipids that become glycated due to exposure to sugars. AGEs accumulate in retinal tissue, causing inflammation, oxidative stress, and vascular damage [13].

Furthermore, hyperglycemia induces a pro-inflammatory state of the retina. The upregulated inflammatory cytokines and chemokines attract immune cells, contributing to vascular leakage, capillary closure, and neovascularization [14]. On the other hand, hypoxia resulting from capillary occlusion stimulates the production of vascular endothelial growth factor (VEGF), an effective angiogenic factor. VEGF promotes the growth of new blood vessels (neovascularization) and increases vascular permeability, leading to macular edema and hemorrhage [15].

Moreover, increased adhesion of leukocytes (white blood cells) to the retinal capillary endothelium leads to capillary occlusion and further exacerbates retinal ischemia and inflammation [16]. As a result of hyperglycemia, the induced damage to pericytes, the contractile cells that wrap around the endothelial cells of capillaries, leads to capillary instability and leakage. Therefore, thickening of the basement membrane, a key structural component of blood vessels, further impairs vascular function [17].

Prevalence of DR

The prevalence of DR varies widely across different populations and geographic regions, influenced by factors such as diabetes incidence, duration of disease, and healthcare access [18].

According to the International Diabetes Federation (IDF), approximately 463 million adults worldwide had diabetes in 2019, with this number projected to rise to 700 million by 2045. Estimates for US indicate that approximately one-third of these individuals may exhibit some form of DR, and 10% may present a form of vision-threatening DR (VTDR), which includes PDR and DME [19].

DR is most prevalent in regions with high diabetes prevalence and limited access to healthcare. For instance, studies have reported higher rates of DR in South Asia, the Middle East, and Sub-Saharan Africa compared to North America and Europe [20,21].

Furthermore, DR prevalence tends to be higher in older individuals probably due to the longer duration of diabetes. Gender differences in DR prevalence are less consistent, with some studies reporting higher rates in men and others finding no significant differences between men and women [22].

Socio-economic factors, including income, education, and access to healthcare, significantly influence the prevalence and severity of DR. Individuals with lower socio-economic status are at higher risk of DR due to factors such as poor glycemic control, lack of access to regular eye examinations, and delayed treatment [23].

Risk factors for DR

Quite a lot of risk factors are associated with DR development and progression. The risk of developing DR increases with the length of diabetes progression. Most patients with type 1 diabetes will develop DR within 20 years after diagnosis, while about 60% of patients with type 2 diabetes will develop DR within the same period [24].

Poor glycemic control is also a major risk factor for diabetes. Higher levels of glycated hemoglobin (HbA1c), a marker of long-term blood glucose control, are associated with an increased risk of DR as well [25]. High blood pressure contributes to DR progression by exacerbating vascular damage and increasing the risk of macular edema [26].

Elevated levels of serum lipids, particularly triglycerides and low-density lipoprotein cholesterol (LDL), are likewise associated with increased risk of DR and macular edema [27]. Endothelial dysfunction and inflammation, possibly due to shared pathogenic mechanisms, link diabetic kidney disease and increased risk of DR [28].

According to recent studies, obesity is also associated with insulin resistance and poor glycemic control, both increasing the risk of DR [29]. In addition, pregnancy can accelerate the progression of DR, particularly in women with pre-existing diabetes and poor glycemic control [30].

Furthermore, susceptibility to DR is influenced by genetic predisposition. Certain genetic polymorphisms have been associated with an increased risk of DR [31]. As stated by Higashi, smoking exacerbates oxidative stress and inflammation, increasing the risk of DR [32]. Furthermore, Spanakis and Golden concluded that certain ethnic groups, such as African Americans, Hispanics, and South Asians, have a higher prevalence of DR compared to Caucasians, likely due to genetic and socio-economic factors [33].

The risk factors for DR are summarized in Table 1.

**Table 1:** Risk Factors Causing Diabetic Retinopathy. DR: diabetic retinopathy; LDL: low-density lipoprotein

Risk Factor	Description
Diabetes Duration	Longer diabetes duration increases DR risk, with nearly all type 1 diabetics developing DR within 20 years.
Poor Glycemic Control	High levels of glycated hemoglobin (HbA1c) are strongly associated with an elevated risk of DR.
Hypertension	High blood pressure exacerbates vascular damage and the risk of macular edema.
Dyslipidemia	Elevated triglycerides and LDL cholesterol levels are linked to DR and macular edema.
Diabetic Kidney Disease	Endothelial dysfunction and inflammation link kidney disease to a higher DR risk.
Obesity	Associated with insulin resistance and poor glycemic control, increasing DR risk.
Pregnancy	Accelerates DR progression, especially with pre-existing diabetes and poor glycemic control.
Smoking	Increases oxidative stress and inflammation, worsening DR.
Ethnic and Genetic Factors	Higher prevalence of DR in African Americans, Hispanics, and South Asians due to genetic and socioeconomic factors.
Socioeconomic Status	Limited access to healthcare, poor glycemic control, and delayed treatment increase DR severity.

Definition and measurement of the AL

As it is well known, the AL is a critical anatomical parameter that influences the refractive status of the eye, playing a significant role in conditions such as myopia (nearsightedness), hyperopia (farsightedness), and emmetropia (normal vision). The AL is a major determinant of the eye’s optical power and, consequently, visual acuity [34].

Accurate measurement of the AL is essential in various clinical and research settings, particularly in the diagnosis and management of refractive errors, the calculation of intraocular lens (IOL) power for cataract surgery, and the assessment of ocular conditions such as glaucoma and retinal detachment. Several methods may be used to measure AL, including A-scan ultrasonography, optical coherence biometry, swept-source optical coherence tomography (SS-OCT), and magnetic resonance imaging (MRI) [35].

Factors influencing the AL

Numerous genetic, environmental, and physiological factors influence the AL. A study showed that the AL is highly heritable, with a significant genetic contribution to its variation. Studies in families and twins have demonstrated that genetic factors play a major role in determining the AL [36].

Genome-wide association studies have identified specific genetic variants associated with the AL and myopia. These include genes involved in ocular growth and development [37].

Martínez-Albert et al. [38] suggest that increased near-work activities, such as reading and use of digital devices, have been associated with a longer-term AL and myopia development. Studies have also shown that higher levels of outdoor activity, possibly due to increased exposure to sunlight and distance vision, may offer protection against myopia and longer-term AL [38].

In 2022, Lee and Mackey published a review of Raine study, claiming that the AL increases rapidly during childhood and adolescence, as part of normal ocular development. Most of this growth occurs before age 6, with slower elongation continuing into the teenage years. During maturity, the AL remains relatively stable. However, certain conditions, such as high myopia, can result in continued elongation into adulthood [39].

Individuals with myopia generally have a longer AL compared to those with emmetropia or hyperopia. High myopia (greater than -6 diopters) is particularly associated with significant elongation of the eyeball. Hyperopic eyes tend to have a shorter AL, which results in the focal point of light being behind the retina, causing farsightedness [40].

Rudnicka et al. support that some variations may exist in the AL across different ethnic groups. For example, East Asian populations tend to have higher rates of myopia and longer AL compared to Caucasian populations [41]. In addition, there are slight differences in the AL between men and women, with men generally having a longer AL. However, the clinical significance of this difference is minimal [42]. Certain systemic conditions, such as diabetes, can influence the AL. For instance, fluctuations in blood sugar levels can cause temporary changes in the AL due to osmotic effects on the lens and vitreous [43].

Evidence for the protective association between the AL and DR: insights from clinical research

Extensive research has explored the relationship between the AL, a defining feature of myopia, and DR. These studies suggest that an increased AL may confer a protective effect against the development and progression of DR, likely mediated by structural, metabolic, and inflammatory mechanisms.

Key Clinical Studies Highlighting the Protective Role of the AL

Early investigations by Baker et al. [44] and recent findings by Thakur et al. [45] emphasized a strong protective association between high myopia and DR risk. Baker et al. observed that the absence of myopia significantly heightened DR risk in individuals with susceptible HLA-DR phenotypes, with odds ratios (ORs) of 11.8 for proliferative PDR and 7.4 for NPDR. However, the presence of myopia reduced these ORs to 0.09 for both PDR and NPDR, representing a nearly 100-fold reduction in risk for these high-risk groups. Rand et al. [46] extended these findings to type 2 diabetes, reporting that high myopia was associated with a markedly lower risk of vision-threatening DR (VTDR), with an adjusted OR of 0.18. While no significant association was observed in type 1 diabetes, this disparity highlights the possible influence of disease duration and pathophysiological differences in DR development.

Inverse Relationship Between the AL and DR Risk

A consistent inverse association between the AL and DR risk has been reported across multiple studies. Early work by Klein et al. [47] and recent research by Wang et al. [48] highlighted that individuals with longer AL exhibited lower prevalence and severity of DR. Wang et al. quantified this protective effect, reporting an OR of 0.81 per millimeter increase in AL, even after adjusting for confounding factors such as diabetes duration and fasting blood glucose levels. In contrast, Varma et al. [49] and Xie et al. [50] hypothesized that a shorter AL, indicative of hyperopia, increases the risk of DR by concentrating pro-inflammatory cytokines like VEGF. Supporting this hypothesis, Xu et al. [51] demonstrated that a shorter AL was significantly associated with a higher risk of DR, with an OR of 0.48 per millimeter increase in the AL, reinforcing the idea that axial elongation mitigates DR progression through a structural and biochemical lens.

Broader Population-Level Evidence

Large-scale, population-based studies further solidify the protective role of an increased AL against DR. Yang et al. [52] reported that each millimeter increase in AL corresponded to a 39.5% reduction in PDR risk (adjusted OR 0.605) in patients with long-term diabetes. Pan et al. [53] confirmed these findings, observing that the prevalence of DR decreased significantly for each millimeter increase in AL, further reinforcing the inverse relationship. Ten et al. [54] utilized data from the National Health and Nutrition Examination Survey (NHANES) to show that high myopia, characterized by a longer AL, reduced DR risk by 56% (OR 0.44), with particularly pronounced effects among non-Hispanic Black populations. Moss et al. [55] and Chao et al. [56] focused on the broader population-level impacts of myopia and AL on DR risk. At the beginning, Moss et al., found that myopia correlated with a longer AL, was a protective factor against the progression to PDR in younger-onset diabetic individuals, with an OR of 0.40 [55].

Mechanistic Insights Into AL's Protective Role

Several studies have proposed plausible mechanisms underpinning the protective effects of the AL on DR. Lim et al. [57] suggested that the reduced retinal hypoxia and metabolic demand in highly myopic eyes, characteristic of longer AL, may protect against DR by alleviating key drivers of its pathogenesis. Man et al. [58] expanded on this by showing that a longer AL correlates with decreased retinal oxygen consumption, thereby minimizing retinal hypoxia-a critical factor in DR progression. Lin et al. [59] added that the thinning of retinal veins in highly myopic eyes reduces venular caliber and leakage, further inhibiting DR progression.

Role of Cytokine Regulation in the Protective Effect

The regulation of inflammatory and angiogenic cytokines also plays a significant role. Kulshrestha et al. [60] found a negative correlation between the AL and placental growth factor (PLGF) levels in NPDR patients, supporting the hypothesis that axial elongation mitigates cytokine-mediated retinal damage. Similarly, Hong et al. [61] demonstrated that eyes with a longer AL had lower VEGF levels in the aqueous humor, suggesting that axial elongation decreases VEGF secretion, thereby reducing DR-associated angiogenesis and inflammation.

Meta-Analyses Strengthening the Evidence

Meta-analyses provide robust quantitative evidence of AL's protective role. Fu et al. [62] pooled data from 11 studies and found a statistically significant 25% reduction in DR risk per millimeter increase in the AL (OR 0.75, 95% CI: 0.65-0.86, p < 0.001). Additionally, myopic eyes exhibited a 30% lower risk of DR compared to non-myopic eyes (OR 0.70, 95% CI: 0.58-0.85, p < 0.001). The meta-analysis demonstrated high sensitivity and minimal publication bias, underscoring the reliability of these findings. Similarly, Wang et al. [63] analyzed nine studies and reported a pooled OR of 0.79 per millimeter increase in the AL, with an even greater protective effect for VTDR (OR 0.70, 95% CI: 0.60-0.82, p < 0.000). These findings emphasize the robust and consistent nature of the association.

Linking the AL and DR

In the last few years, the likely protective effect of a longer AL against DR has attracted considerable research interest. Proposals to explain this association have focused on the relationship between the AL and retinal vascular density, retinal blood flow, and ocular biomechanics.

Reduced Retinal Vascular Density Hypothesis

An important hypothesis posits that a larger AL is associated with a reduction in retinal vascular density, which could lower the risk of DR. According to Khan et al. [64], this reduction in vascular density may decrease the overall exposure of retinal tissues to hyperglycemic damage, thereby reducing the likelihood of DR development. As the AL increases, the retinal surface area expands, leading to a dilution effect on the retinal vasculature. This expansion results in fewer blood vessels per unit area, potentially limiting the number of capillaries and microvessels exposed to hyperglycemia-induced damage, such as capillary basement membrane thickening, pericyte loss, and microaneurysm formation.

With reduced vascular density, the metabolic demand of retinal tissues may also decrease. This lower metabolic activity could make retinal cells less vulnerable to the metabolic disturbances often associated with diabetes, including oxidative stress and inflammatory processes. Additionally, fewer blood vessels in the retina might reduce potential sites for inflammation and leukostasis, both of which play significant roles in DR pathogenesis [57,64,65].

Mechanistically, Fu et al. proposed that the protective effects of myopia and increased AL stem from structural and functional adaptations in myopic eyes. These include retinal thinning and elongation, which are thought to reduce metabolic demand and oxygen consumption. By mitigating hypoxic conditions, a key driver of DR progression, these changes may reduce the risk of vascular dysfunction. Furthermore, the increased ocular volume associated with a longer AL could dilute inflammatory mediators, such as VEGF, which are strongly implicated in the pathogenesis of DR. This dilution effect may decrease the concentration of pro-inflammatory and pro-angiogenic factors in the retina, thereby offering additional protection [62].

Importantly, Fu et al. [62] emphasized that axial elongation, rather than refractive error alone, plays a central role in this protective mechanism. Their findings align with other studies that highlight reduced metabolic retinal activity and altered blood flow dynamics in eyes with a longer AL, further supporting the hypothesis of a protective relationship between axial elongation and DR.

Altered Retinal Blood Flow Hypothesis

Shimada et al. [66] hypothesized that changes in retinal blood flow dynamics associated with a longer AL may play a protective role against the development of DR. In eyes with an increased AL, reduced retinal blood flow could decrease oxygen and metabolic demands in retinal tissues. This reduction in metabolic activity might lower the susceptibility of retinal tissues to vascular damage, which is commonly exacerbated by hyperglycemia.

Yang and Koh [67] further demonstrated that myopic eyes with longer AL exhibit altered hemodynamics, including lower perfusion and blood velocity compared to emmetropic or hyperopic eyes. This difference in blood flow dynamics suggests that retinal tissues in myopic eyes may experience less exposure to hyperglycemia-induced oxidative stress and inflammation, both of which are critical drivers of DR pathogenesis.

Rudraraju et al. [68] provided additional insights, indicating that reduced blood flow in longer eyes may limit glucose delivery to the retina. While this reduction may initially seem detrimental, it could, in fact, alleviate hyperglycemic stress on retinal endothelial cells and mitigate the overproduction of AGEs and ROS. These biochemical changes are key contributors to vascular dysfunction in DR.

Moreover, Wright et al. [69] suggested that altered retinal blood flow dynamics in myopic eyes with a longer AL might help maintain the integrity of the blood-retina barrier (BRB). A stable BRB reduces vascular permeability, thereby minimizing the risk of fluid leakage and macular edema-both characteristic features of advanced DR. This mechanism underscores the potential protective role of altered blood flow in mitigating DR progression in eyes with a longer AL.

Ocular Biomechanics Hypothesis

The structural and biomechanical properties of the eye, which vary with the AL, may play a crucial role in influencing the development of DR. Barot et al. [70] suggested that longer eyes experience reduced mechanical stress on retinal capillaries due to their altered shape and biomechanics. This reduction in stress might decrease the likelihood of microvascular damage, a key precursor to DR.

As the AL increases, the retina and choroid undergo stretching and thinning. These structural changes may redistribute and reduce the mechanical forces acting on the delicate retinal capillaries, potentially protecting them from mechanical deformation or rupture. Mohite et al. [71] elaborated on this by demonstrating that decreased stress levels on capillaries in elongated eyes lower the probability of capillary damage and the formation of microaneurysms-one of the earliest and most characteristic features of DR.

Additionally, the elongation and thinning of ocular structures in myopic eyes may influence the diffusion of metabolic substrates, such as oxygen and glucose, as well as the clearance of waste products. This altered diffusion pattern could modify the retinal microenvironment, reducing hypoxic and hyperglycemic stress on retinal cells. Mohite et al. [71] proposed that such a microenvironment is less favorable for the biochemical and cellular processes that drive DR progression, including oxidative stress, inflammation, and endothelial dysfunction.

Together, these biomechanical and structural adaptations in longer eyes may provide a degree of protection against the microvascular complications associated with DR by minimizing mechanical and metabolic stress on retinal tissues.

Limitations and controversies

Despite the promising implications of the AL in DR management, several limitations and controversies need to be addressed. One significant issue is the presence of confounding factors such as age and the duration of diabetes. Both factors are independently associated with higher DR risk and can influence study results, complicating the interpretation of the relationship between the AL and DR. Alshahrani et al. concluded that poor glycemic control, a well-established risk factor for DR, adds another layer of complexity, as variations in HbA1c levels across study populations can confound the relationship [72].

Ethnic and genetic differences also play a role in this relationship, further complicating the interpretation of results across diverse populations [37,41]. Additionally, methodological challenges limit the current body of research. Many studies are cross-sectional, which limits the ability to establish causality between the AL and DR. Longitudinal studies are needed to confirm whether a longer-term AL indeed has a protective effect over time. Measurement variability, due to differences in AL measurement techniques and criteria for diagnosing DR, introduces further inconsistencies in study outcomes. Moreover, limited sample sizes and a lack of diversity in study populations can affect the generalizability of findings. Larger, multi-ethnic studies are necessary to validate these results.

Ongoing debates also exist regarding the mechanistic understanding of how the AL influences DR risk. Despite the proposal of several hypotheses, the precise biological mechanisms remain ambiguous, underscoring the necessity for additional research. Additionally, there is ongoing debate about the practical utility of incorporating AL measurements into routine clinical practice for DR risk assessment. Further evidence is required to demonstrate the cost-effectiveness of this approach and its impact on patient outcomes [64-69]. The presence of other ocular conditions, such as cataracts or glaucoma, which can influence AL measurements and confound the relationship with DR, adds another layer of complexity that future studies must account for [73].

Clinical implications and future directions

The inverse relationship between the AL and DR has profound clinical implications, particularly in the screening and monitoring of diabetic patients. Patients with a longer AL, who are at a lower risk of developing DR, may require fewer frequent retinal examinations. This personalized approach can significantly enhance the efficiency of healthcare delivery. Conversely, patients with a shorter AL, who are at higher risk, may benefit from more frequent monitoring to detect and manage DR at an earlier stage, potentially improving patient outcomes.

Incorporating AL measurements into routine ophthalmic assessments could also improve DR risk stratification's accuracy. By improving risk assessment, we can focus preventive and therapeutic efforts on individuals who are most at risk. The development of predictive models that include AL as a variable further underscores the potential of AL measurements to inform risk valuation. These models, when combined with other known risk factors such as the duration of diabetes, HbA1c levels, and blood pressure, can provide a more comprehensive evaluation of DR risk. Ultimately, this better risk stratification can lead to personalized treatment plans that improve patient outcomes.

Personalized treatment approaches based on the AL are another critical area of clinical application. To prevent DR progression, patients with a shorter AL may benefit from more aggressive interventions like stricter glycemic control or early laser treatment. On the other hand, patients with a longer AL might benefit from a more conservative approach, reducing the risk of overtreatment and associated complications. Understanding the mechanisms by which longer AL confers protection against DR could also lead to the development of novel therapies that mimic these protective effects, offering new treatment options.

Given the limitations and controversies outlined, several recommendations for future research emerge. Longitudinal studies that follow individuals over time are crucial for establishing causativeness and better understanding the protective role of a longer AL against DR. Additionally, mechanistic studies that investigate the biological pathways linking AL and DR can provide insights into potential therapeutic targets. This research should utilize advanced imaging and molecular techniques to uncover the underlying mechanisms at play.

The inclusion of diverse populations in future studies is also essential. Multi-ethnic cohorts are necessary to validate findings across different ethnic and genetic backgrounds, ensuring the generalizability of results. Technological advancements in AL measurement, such as the development of more precise and non-invasive biometry techniques like swept-source optical coherence tomography can improve the accuracy of AL measurements and facilitate large-scale epidemiological studies. The availability of portable AL measurement devices could expand access to accurate measurements in a variety of clinical settings, including remote or underserved areas.

The potential for developing new therapeutic strategies based on the protective mechanisms associated with a longer AL is another promising area for future research. These insights could lead to the development of therapies that modulate retinal vascular density or improve retinal blood flow. Additionally, understanding the protective mechanisms of longer AL may lead to the development of pharmacological or gene therapies that replicate these effects, offering new treatment options for DR. Early identification of high-risk individuals based on AL measurements could also facilitate the implementation of preventive interventions, potentially delaying or preventing the onset of DR.

## Conclusions

In conclusion, the relationship between the AL and DR represents a compelling area of research with significant clinical implications. While epidemiological evidence supports an inverse relationship between the AL and DR, suggesting that a longer-term AL may procure protection against the development and progression of DR, several limitations and controversies remain. Addressing these challenges through future research is critical, particularly with a focus on longitudinal studies, mechanistic investigations, and the inclusion of diverse populations. Technological advancements in AL measurement, as well as the development of new therapeutic strategies based on these insights, hold promise for improving the management and outcomes of DR. By integrating AL measurements into routine clinical practice and exploring innovative therapies, healthcare providers can better tailor their approaches to individual patient needs, ultimately enhancing the quality of care for individuals with diabetes.
